# A cost-precision model for marine environmental monitoring, based on time-integrated averages

**DOI:** 10.1007/s10661-017-6064-6

**Published:** 2017-06-25

**Authors:** Ulf Båmstedt, Sonia Brugel

**Affiliations:** 1 Umeå Marine Sciences Centre, Umeå University, Norrbyn, SE-905 71 Hörnefors, Sweden; 2 Department of Ecology and Environmental Science, Umeå University, SE-901 87 Umeå, Sweden

**Keywords:** Environmental surveys, Cost of precision, Optimal allocation, Seasonal variability, Marine environments, Coastal ecology

## Abstract

Ongoing marine monitoring programs are seldom designed to detect changes in the environment between different years, mainly due to the high number of samples required for a sufficient statistical precision. We here show that pooling over time (time integration) of seasonal measurements provides an efficient method of reducing variability, thereby improving the precision and power in detecting inter-annual differences. Such data from weekly environmental sensor profiles at 21 stations in the northern Bothnian Sea was used in a cost-precision spatio-temporal allocation model. Time-integrated averages for six different variables over 6 months from a rather heterogeneous area showed low variability between stations (coefficient of variation, CV, range of 0.6–12.4%) compared to variability between stations in a single day (CV range 2.4–88.6%), or variability over time for a single station (CV range 0.4–110.7%). Reduced sampling frequency from weekly to approximately monthly sampling did not change the results markedly, whereas lower frequency differed more from results with weekly sampling. With monthly sampling, high precision and power of estimates could therefore be achieved with a low number of stations. With input of cost factors like ship time, labor, and analyses, the model can predict the cost for a given required precision in the time-integrated average of each variable by optimizing sampling allocation. A following power analysis can provide information on minimum sample size to detect differences between years with a required power. Alternatively, the model can predict the precision of annual means for the included variables when the program has a pre-defined budget. Use of time-integrated results from sampling stations with different areal coverage and environmental heterogeneity can thus be an efficient strategy to detect environmental differences between single years, as well as a long-term temporal trend. Use of the presented allocation model will then help to minimize the cost and effort of a monitoring program.

## Introduction

Ongoing marine environmental monitoring programs, following the European Water Framework Directive, are designed to detect long-term trends over several years and to classify the environmental state into one of five ecological quality classes, ranging from bad to high quality (Heiskanen et al. 2004; Carstensen 2007). Marine environmental monitoring programs usually include only very few stations which are supposed to be representative of large areas. Carstensen (2007) used data from Limfjorden in Denmark over the period 1989–2004 and showed that 1291 samples of chlorophyll *a* was needed to define the quality classification within 5% with 80% power when a single station was monitored. Ongoing monitoring programs usually only include a small fraction per year of this number, e.g., one per month, which then would require more than 100 years to achieve sufficient data for a corresponding classification. Carstensen (2007) discusses methods to reduce the residual error by using for example seasonal adjustment models and covariates in the statistical analyses. Environmental data usually also needs to be transformed to achieve approximately normal distribution and sequential sampling over time tends to be autocorrelated (e.g., Priestley 1982), which causes problems in the statistical treatments. We show in the present paper that the sampling program and statistical procedures can be feasible when the goal is to only get an estimate of the annual or seasonal average of a variable, without an evaluation of within-year fluctuations. Such a program will be suitable for comparing the environmental condition between single years. This is highly needed in environmental research, where there is a common problem to find logic explanations for changes in for example primary production (e.g., Rydberg et al. 2006), zooplankton abundance (e.g., Moller et al. 2015), and recruitment of fish (e.g., Pecuchet et al. 2015) between consecutive years in the ecosystem.

Since aquatic environments are three-dimensional, more so than terrestrial environments, it might require measurements along a depth scale, dependent on the aim of the study. The pelagic habitat and its ecosystem are by definition not stationary in space, thereby introducing variability in a fixed sampling station due to e.g., migration of organisms and water mass exchange.

Since economy often constrains the scope of planned monitoring programs, it is necessary to optimize the design in a way that will give sufficient precision of the estimated parameters for the available budget. The cost for research ships for offshore operations may reach 10,000 € or more per day, making it especially important to optimize the field program. Our presented allocation model can be a guideline to design such a sampling program based on a given budget.

Technical developments in moored environmental sensor buoys, autonomous underwater vehicles (AUVs) and free-floating sensor buoys, and wireless communication have greatly improved the possibilities of efficiently monitoring environmental variables (e.g., Bogue 2011, Marcelli et al. 2014, Xu et al. 2014). However, there are still constraints, especially in their ability to measure biological variables (Gibbs 2012), and their accuracy is often lower than laboratory based analyses can provide. However, improvements in biological sensors (Moustahfid et al. 2012) have opened up for including more biological variables on the different sensor platforms. But a remaining problem is their need of regular maintenance, e.g., due to biofouling and power limitations.

## Material and methods

### The model

We can define a linear equation for the cost of a survey as given below.

1 $$ \mathrm{Cost}={N}_{\mathrm{time}}\times {N}_{\mathrm{space}}\times \left[ A/ n+\sum \left({m}_{\mathrm{i}}\right)\right]+ B $$

where the terms are defined below:

*N*_time_ = number of surveys per station over the year

*N*_space_ = number of stations per survey

*A* = cost per day for research ship

*n* = average number of stations per cruise day

*m*_i_ = cost for sampling and analysis of variable i

*B* = labor costs necessary for field and laboratory work, data compilation and reporting, and overhead cost and cost of other administrative work

The model can of course be made more detailed, including for example costs for land transportation, overtime costs, instrumentation, and equipment, and such factors can easily be included to get a good representation of the actual costs in each specific case. If they are proportional to the number of stations included, they add to the factors within the brackets in Eq. (1), otherwise they add to the factor *B* in the end of the equation.

**Fig. 1:**
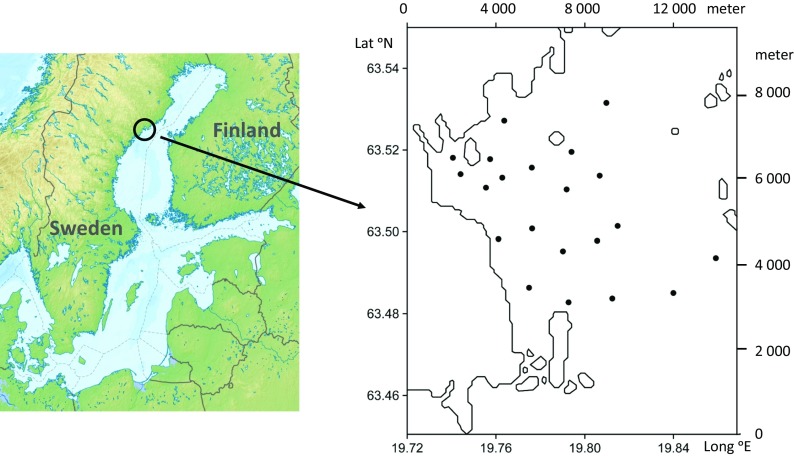
Map showing the geographical position of the 21 sampling localities. Axes giving the latitude (°N) and longitude (°E) as well as the distance in meter

The model is based on the variance between time-integrated averages of the sampling stations in a defined area (Fig. 1). The annual average for a station is calculated as ∑(*X*_i_ ∗ *t*_i_) / ∑*t*_i_, where *X*_i_ is the measured value of the variable at the *i*th sampling time and *t*_i_ is the corresponding time interval as defined by the time midpoints between measurements. In order to find an optimal number of samples over the year that should be used, we need information on how sampling frequency governs the estimated annual average and variance. We suggest to do a 1-year pilot study on one or a few key variables with measurements every week in all sampling stations. From this dataset, we can do a stepwise reduction in sampling frequency and calculate annual averages for each station and thereby annual average and variance for the area under investigation. We used 5% as maximum deviation from the result of highest sampling frequency as the critical level, i.e., the lowest frequency that gave results within ±5% of highest frequency should be used. This number is then the factor *N*_time_ in Eq. (1).

With a pre-defined budget, we can calculate the precision we will achieve. First, we calculate *N*_space_ by using the pre-defined *N*_time_ in the equation below:

2 $$ {N}_{\mathrm{space}}=1/{N}_{\mathrm{time}}\times \left[\mathrm{Cost}- B\right]/\left[ A/ n+\sum \left({m}_{\mathrm{i}}\right)\right]. $$

The relationship between the 95% confidence interval for the estimated arithmetic mean (CI%) and the underlying coefficient of variation in the population (CV%) is given by the formula CI% = *t*_0.05_ × CV% × (*N*)^−0.5^, where *t*_0.05_ is given from a table of Student’s *t* statistic. Therefore, the precision as 95% confidence interval is given by Eq. (3) below:

3 $$ \mathrm{CI}\%={t}_{0.05}\times \mathrm{CV}\%\times {\left[1/{N}_{\mathrm{time}}\times \left(\mathrm{Cost}\hbox{--} B\right)/\left( A/ n+\sum \left({m}_{\mathrm{i}}\right)\right)\right]}^{-0.5} $$

The description above relates to a single variable, whereas a monitoring program usually includes several variables, each of them with their specific variability in time and space. As long as we have estimated their CV%, the model can also provide the precision as CI% for them, either through the formula CI% = *t*_0.05_ × CV% ∗ (*N*)^−0.5^ or by using a spreadsheet or statistical software to directly calculate CI% through input of CV%, *N*, and confidence level (here 95%, i.e., *α* = 0.05).

### Material for model tests

For all statistical calculations and tests, we used the statistical software package Systat 13.1 (Systat software Inc., www.systat.com). Weekly vertical profiles of environmental variables were taken at 21 stations in an estuarine system in northern Bothnia Sea between 63.47°–63.52° N and 19.74°–19.86° E (Fig. 1) from June to December 2013. A logging instrument (Aanderaa SeaGuard, *See* www.aanderaa.com) with six different sensors were slowly lowered from the surface to the bottom with a measuring frequency of 1 per second. Sensors measured salinity, temperature, light (PAR, photosynthetic active radiation, 400–700 nm spectrum), oxygen saturation and concentration, chlorophyll *a*, and CDOM (chromatic dissolved organic material). The light profiles were used to calculate the light extinction coefficients (*k* in the equation *I*_d_ = *I*_0_ ∗ *e* ^(−k ∗ d)^, where *I*_d_ is the irradiance at *d* meter depth, *I*_0_ is the irradiance at the surface, and *k* is the slope in the exponential equation), whereas average values for 0–3-m depth profiles were used for the other variables. For each of the 21 stations, we calculated the arithmetic average for the whole period, and these 21 values were used to calculate the time-integrated arithmetic average and coefficient of variation, CV%, for each variable. These average CV% values for the different variables were used to calculate the necessary sample size to detect a 5% change, using power analysis at 95% significance level and a power of 80% (*α* = 0.05; *β* = 0.2).

The procedure of only using the time-integrated average of each station can be seen as pooling over time of measurements, and the problems of autocorrelation, seasonal trends, and non-normal distribution over time have no impact on the results. Test of normality of these temporal averages of the 21 stations, using Shapiro-Wilk test statistic (Shapiro and Wilk 1965) showed no significant deviation from normality for the six variables. In order to show how this procedure reduces variability in the estimates, we made calculations on temporal variability of each station and spatial variability at each time point. This was done for original data as well as for log-transformed data (improving normal distribution).

In order to define an optimal number of samples for the time-integrated average that should give a high precision with lowest cost, we calculated the arithmetic average and 95% confidence interval with sequentially reduced number of samples, going from one per week (24 samples) and down to one per 6 weeks (four samples). The mean and confidence interval were based on all 21 stations and standardized to show the result for 24 time points as 100%, in this context defined as “true value.” We used ±5% as maximum acceptable deviation from the true value, i.e., the result for 24 time points.

We used power analysis (one sample *t* test) on the time-integrated averages for the 21 stations in order to define sample size requirements for detecting 5 or 10% change in time-integrated averages between years. We then used our estimated time-integrated averages and CV% values as input, assuming the same CV% for 2 hypothetical years, and presented the result as graphs of statistical power versus sample size.

## Results

### Weekly vertical sensor profiles

**Fig. 2:**
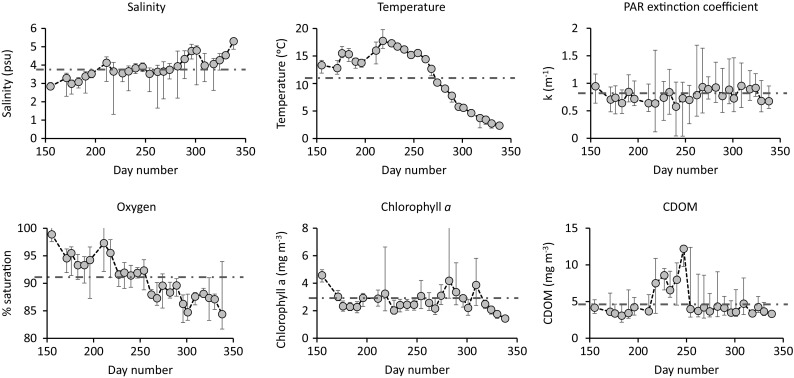
Arithmetic mean and range of results integrated over 0–3-m depth for the 21 stations with a panel for each of the six variables over the time span from day 155 to day 338. Overall arithmetic mean indicated by the *horizontal dotted line*

Fig. 2 shows arithmetic mean and range of results, integrated over 0–3-m depth, for the 21 stations with a panel for each of the six variables over the time span from day 155 to day 338, i.e., early June to early December. Regular trends are shown by salinity, slowly increasing over time, temperature first increasing then after day 220 steadily decreasing, and for oxygen which decreases over the whole period. The other three variables show irregular variability, except for CDOM, with peaked values between day 218 and 247. Table 1 summarizes the results for all six variables included. A Shapiro-Wilk test indicated a normal distribution of the time-integrated averages for the 21 stations included, and the average CV% for the six variables ranged from 0.55 (oxygen) to 12.36% (PAR extinction coefficient). The relatively small CV% values indicated a rather narrow distribution of time-integrated average values among the 21 stations, although the total range in recorded values was high (*See* Fig. 2, Table 1). The average CV% and range from single time points (CV% of 21 stations at each of the 24 time points) is presented in Table 2, both based on original data and on log-transformed data. Similarly, CV% and range from individual stations over the whole investigation period are also presented (Table 2). Except for salinity variability over stations, CV% based on individual time points is at least 2–3 times higher than those based on time-integrated averages, with the ranges reaching ten times higher values. Similar results are also shown for variability over time (Table 2). The sample size required for detecting a 5% difference at a given time point or at a given station would then be high in most cases (*See* Table 2).

**Table 1:** Time-integrated arithmetic average, range, and coefficient of variation of time-integrated average (given as CV% = SD / mean × 100%) within the area investigated based on all 21 stations and all 24 time points. The equation to calculate the precision (CI%) of the annual mean is also given

Variable	Arithmetic averages	Range	CV% of averages	CI% equation
Salinity (g dm^−3^)	3.90	2.20–5.40	6.21	12.48 × *N* ^−0.5^
Temperature (°C)	11.19	1.91–18.37	0.97	1.95 × *N* ^−0.5^
PAR extinction coefficient (*k*, m^−1^)	0.77	0.04–1.69	12.36	24.83 × *N* ^−0.5^
Oxygen saturation (%)	90.44	81.69–106.97	0.55	1.10 × *N* ^−0.5^
CDOM (μg dm^−3^)	4.90	2.18–26.46	10.83	21.76 × *N* ^−0.5^
Chlorophyll *a* (μg dm^−3^)	2.64	1.38–8.18	4.76	9.56 × *N* ^−0.5^

**Table 2:** Estimates of time-integrated arithmetic average of different variables, mean and range of coefficient of variation (CV% = SD / mean × 100), and sample size necessary to detect a 5% change with a power of 80% and a significance level of 95% (*α* = 0.05; *β* = 0.2). Estimates based on the original values from 21 stations and 24 sampling occasions. “*Over time*” means that the parameters are based on the spatial average of the 21 stations at each sampling occasion, i.e., average of 24 CV% values. “*Over stations*” means that the parameters are based on the temporal average for each station over the 24 time points, i.e., average of 21 CV% values. “*Normal*” means that original data from each individual station and time point has been used, and “*log-normal*” means that log transformed data was used

Definition	Statistic	Salinity	Temperature	Ext. coeff.	Oxygen	CDOM	Chlorophyll
Over time							
Normal	Average	3.90	11.23	0.78	90.57	4.89	2.68
	Mean CV%	16.18	46.21	25.82	4.18	50.63	29.51
	CV% range	13.78–29.84	43.28–49.76	17.58–44.03	2.44–5.52	36.35–88.57	17.41–45.61
	Sample size	15	669	211	8	806	275
Log-normal	Average	3.84	9.55	0.74	90.49	4.47	2.57
	Mean CV%	29.34	19.76	191.38	1.15	33.52	51.13
	CV% range	28.46–46.50	16.53–21.58	134–342	1.02–1.22	23.48–38.31	47.50–53.78
	Sample size	272	125	>10,000	3	355	822
Over stations							
Normal	Average	3.90	11.27	0.78	90.87	4.87	2.72
	Mean CV%	6.24	3.68	24.42	1.26	24.23	12.52
	CV% range	1.59–18.47	0.68–9.88	11.66–63.68	0.38–3.93	3.38–110.66	2.80–37.65
	Sample size	85	7	189	3	187	52
Log-normal	Average	3.83	11.27	0.75	90.83	4.74	2.69
	Mean CV%	31.00	13.85	189.61	1.12	29.06	44.40
	CV% range	19.28–36.86	5.83–44.09	123–440	1.15–1.16	8.67–43.55	23.18–71.59
	Sample size	304	63	>10,000	3	267	621

**Fig. 3:**
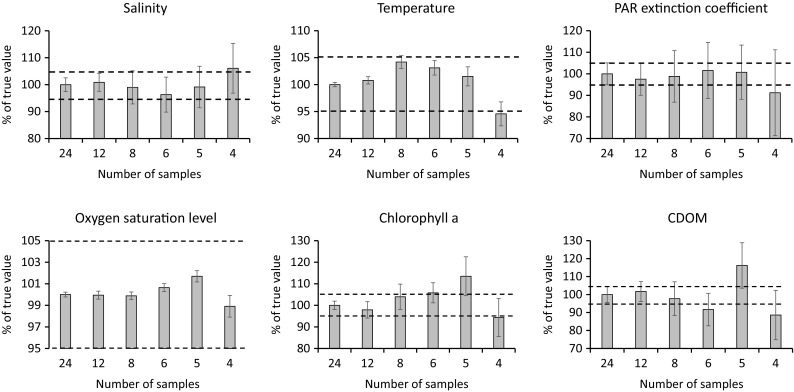
Result on the calculated time-integrated average and 95% confidence interval (thin vertical lines) when reducing the number of time points from weekly (24 time points) to sequentially 12, 8, 6, 5, and 4. Results given as % of weekly sampling. Horizontal lines denote 95 and 105%

The result on the calculated time-integrated average when reducing the number of time points in the original series of 24 to sequentially 12, 8, 6, 5, and 4 is shown in Fig. 3. If the critical level is defined as ±5% deviation from the reference value (result with 24 time points), we see that oxygen stay within that level with all sampling frequencies tested. Salinity, temperature, and PAR extinction coefficient all gave mean values within the 5% limit for all but the lowest sampling frequency, whereas chlorophyll *a* and CDOM were acceptable when using 12 and 8 time points.

### The optimal allocation model

We have used the results on variability, expressed as CV%, from the weekly vertical profiles of the six variables from 21 stations (*See* Table 1) in the model. The relationship between precision, given as CI%, and number of stations included is given in Table 1. By using these equations, we can see that oxygen and temperature can reach a precision better than 2% already with two stations included, whereas the extinction coefficient and CDOM require 25, respectively, 19 stations to reach a precision of 5%.

The cost for a seasonal survey, when requiring a given precision for a given parameter, can be calculated according to Eq. (1). The other included variables achieve different precisions related to their CV%. In our example, we have used the following constants in Eq. (1):

$$ A=1; B=20; n=3;\sum \left({m}_{\mathrm{i}}\right)=0.1. $$

**Fig. 4:**
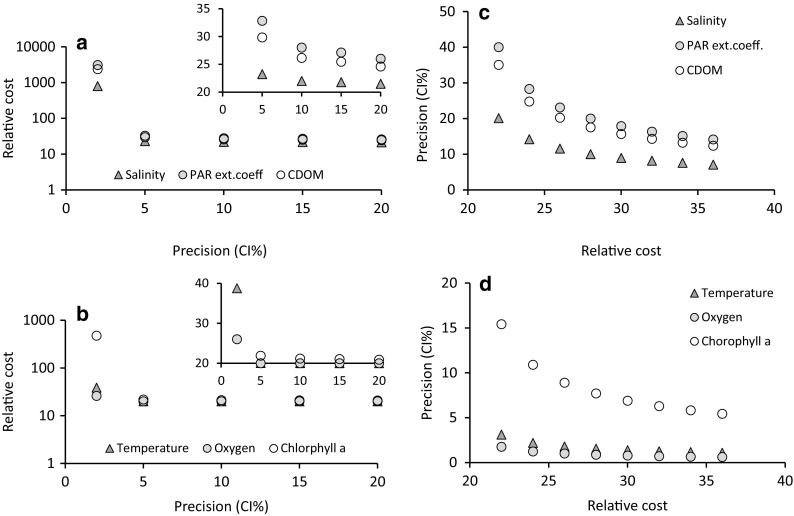
Relative cost in relation to precision of the estimated time-integrated average for the six variables (a and b) and the precision achieved with a fixed budget (c and d). Inserted graphs in a and b display a higher resolution in part of the precision range

When using the model, the real costs and currency for the different constants should be used. Fig. 4A and B shows the relationship between relative total cost and precision in the estimated time-integrated average for the six variables. Salinity PAR, CDOM, and chlorophyll *a* would cost between 22 and 94 times more by increasing the precision from 5 to 2%, whereas temperature and oxygen would cost only 1.9, respectively, 1.3 times more with the same precision improvement. The graphs also show that cost reduction through a further reduction in the precision above 5% would be marginal. If we have a pre-defined budget and want to see which precision it provides, we use Eq. (3). With the same constants used in the previous example, we can see the relationship between precision and the level of a fixed budget in Fig. 4C and D. To reach a precision of at least 20% for all six variables, we need a budget of 28 relative units, and with that budget, temperature and oxygen, which are the variables with highest precision, reach 1.4, respectively, 0.9%.

**Fig. 5:**
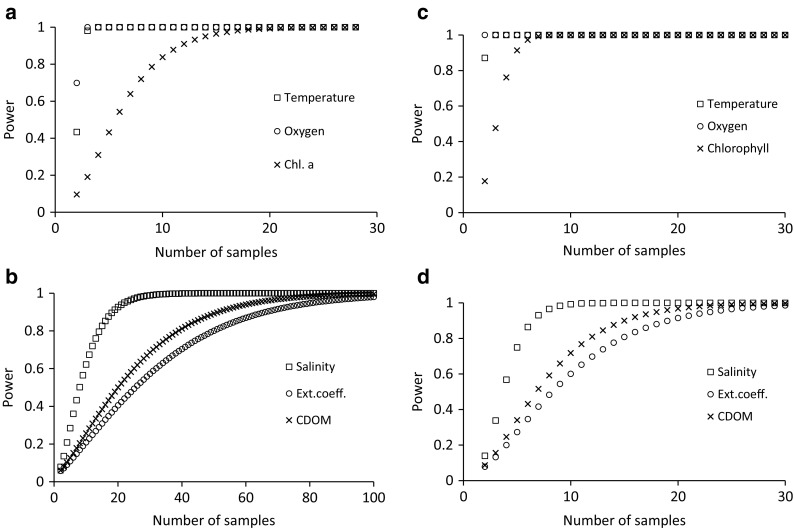
Relationship between sample size (number of stations per cruise) and power to detect 5% change (a, b) or 10% change (c, d) in time-integrated average between different years for the six variables in the study. Total number of samples per year is then this number times number of time points during the year

The power analysis showed that a 5% change in annual mean for oxygen and temperature can be detected with very high power even with a sample size of 2–3 whereas chlorophyll *a* requires a sample size of 10 to reach a power of 0.8 (Fig. 5A). Salinity, CDOM, and PAR extinction coefficient would need sample sizes of 15, 39, and respectively 50 to reach a power of 0.8 (Fig. 5B). If we reduce our requirements to a detection precision of 10%, chlorophyll would require five samples (Fig. 5C), and changes of 10% in salinity, CDOM, and PAR extinction coefficient could be detected with a power of 0.8 by using sample sizes of respectively 6, 12, and 15 (Fig. 5D).

## Discussion

In this paper, we used a dataset of environmental variables covering only half a year. A logic time-unit to use is 1 full year. In high-latitude environments, there is a cyclic variation in the physio-chemical environment on a 1-year time scale. The phytoplankton succession and thereby other trophic levels in the planktonic ecosystem are usually adapted to this and hence show cyclic successions on a 1-year time scale. Therefore, annual means of environmental variables should reflect the average environmental condition for that year. Our dataset is mainly used to illustrate the methodological work and test the model, so the restricted cover of the year that we have used has no implications for the suggestions and conclusions. Furthermore, our dataset is dominated by physical and chemical variables, and even if biological variables in some cases might show higher variability, the basic principles for designing a monitoring program and using the allocation model still holds for such variables.

Inter-annual variability in the marine environment is typical for natural systems and are overlaid the slow changes related to the ongoing climate change occurring over several decades. Spatio-temporal variability within a year is also high, as exemplified in the range of individual records in Table 1 and CV ranges in Table 2. By only using time-integrated averages for each station in the analyses, we minimize effects of seasonal and short-term variations and thereby also reduce variability between stations. Such an effect can be seen in previous published results. One example is the study by Rantajarvi et al. (1998) who recorded chlorophyll *a* along transects from a passenger ferry in the Arkona Sea and the western Gulf of Finland. Data sampled over a 1-year period with sampling frequency varying from one per month to two per week gave a coefficient of variation ranging from 53 to 82% in the Arkona Sea and between 89 and 144% for western Gulf of Finland, as calculated from their Table 1. However, looking at the average chlorophyll concentration for the whole period, it ranged between 2.8 and 3.0 (mean 2.8) μg L^−1^ with a coefficient of variation of 4.4% in the Arkona Sea and between 2.7 and 4.5 (mean 3.9) μg L^−1^ with a coefficient of variation of 11.5% in the western Gulf of Finland. Thus, time-integrating station measurements, ideally over a full year, will reduce variability dramatically between the stations and a high precision in estimates of annual or seasonal parameters for the investigated area can be obtained.

Using pooled data, like we have done through the time integration, has been criticized for loss of important information. This critique is adequate when pooling data from individual organisms like single-fish specimens (Bignert et al. 1994, 2014). In such a case, different co-variates like age, gender, and body-fat level might be of relevance in explaining for example the level of eco-toxins and can be used to divide the population into more homogenous groups. When dealing with plankton ecosystems, each analytical sample is usually based on measurements on hundreds (mesozooplankton) to millions (phytoplankton, bacteria) of individuals and in reality then represent a pooled sample. Furthermore, the result from each time point will be present, and although each single data point should not be used in statistical tests, they will give a good indication of the seasonal variation in the stations.

Since most variables in aquatic environments in general show a vertical gradient, the results also depend on the depth of sampling. Usually variability decreases with depth and sampling only in the surface water will therefore maximize variability. In addition, we usually also find the largest seasonal variability in the surface water, where most of the seasonally variable primary production occurs. In our examples in this paper, we have integrated over 0–3-m depth, and the calculated PAR extinction coefficient showed that 1% of surface level (approximately euphotic zone, e.g., Valiela 1995) on average (±SD) was at 6.04 ± 0.71-m depth, meaning that we covered the upper half of the euphotic zone.

Since labor costs and cost for ship time usually are constraining budgetary factors for running a monitoring program with sufficient precision in estimated parameters, alternatives to the traditional cruise-based programs should be considered. During the last decades, the technical development in remote sensing, environmental sensors and platforms, and wireless sensor networks has been dramatic (e.g., Blondeau-Patissier et al. 2014, Moustahfid et al. 2012, Aguzzi et al. 2011, Albaladejo et al. 2010, Trevathan et al. 2012, Xu et al. 2013, 2014, Thosteson et al. 2009), and such techniques are used for a variety of environmental issues. The largest research program based on autonomous sensor platforms is the global Argo project (*See* http://argo.jcommops.org), where more than 3900 float units are presently spread over the world ocean, each one regularly sending data to host servers. However, as pointed out by Gibbs (2012), sensors for biological quantities and processes are far behind in development compared to sensors for physical and chemical measurements. The consequence of the limited ability to measure different variables, especially biological ones that are also given priority in current international directives (e.g., the Water Framework Directive, e.g., Ferreira et al. 2011), is that autonomous sensor systems still cannot replace traditional ship-based surveys. However, for specific research questions where a high-temporal resolution in data collection is crucial, moored sensor platforms might be the right solution.

Despite the choice of practical design of a monitoring program between traditional ship-based, use of autonomous platforms or combination of both, the use of a cost-precision model would optimize the use of resources. In our presented model, we have used time-integrated averages for each station which, in the studied area, gave normal distributed data and thereby a basis for sound and simple statistics when calculating precision and power. If only a single station is used to represent for example a given area, a water mass or a basin, the sampling size needed to detect a statistically significant change will be high. Data from a station in Limfjorden, Denmark was used by Carstensen (2007) in order to calculate necessary sample size to detect a deviation from a classification boundary level, and for 5% deviation, the necessary sample size ranged from 124 to 3400 (*α* = 0.05, *β* = 0.2) for the seven variables studied, with chlorophyll *a* requiring 1291 samples. If we assume monthly sampling and necessary number of stations to detect a 5% change in annual mean, we should come up with sample sizes between 24 (oxygen) and 504 (PAR extinction coefficient). Chlorophyll *a*, as a potential environmental quality indicator, would require 96 annual samples. If we reduce our requirements to detect a 10% deviation from a boundary level, we would need to take between 24 (temperature and oxygen) and 144 (PAR extinction coefficient) samples, i.e., less than 1/10 of the number presented from a station in Limfjorden by Carstensen (2007).

When using our model, it is necessary to decide which variable or set of variables should define the sampling allocation and which minimum precision we require for them. The sample allocation thereby given will then define the precision obtained for the remaining variables. Alternatively, the model can be used to estimate precision with a pre-defined budget and to calculate the resulting statistical power to detect differences between years. Note that in our examples of the model results, we have used arbitrary constants for cost of ship time, analysis costs, labor costs, number of stations per cruise day, etc. Any changes in these values will change the model results. Based on our own results from the six variables, we have used 12 sampling times in the model. Also, this number might be adjusted, up or down, if available information indicates that. Variables with a clear seasonal trend and where the variance change in parallel with changed level of the measure also require caution in the resource allocation process. Examples of such variables are biological production estimates, e.g., primary production, bacterial production or production at any higher trophic level, as well as abundance of species or taxonomic groups. Allocating more sampling during periods with high levels and high variability of measurements and reducing sampling frequency during periods with low levels and low variability will improve the precision in time-integrated estimates. This usually implies that the period March–September should be sampled more frequently than the period October to February in high northern latitudes, for example with nine of the 12 sampling times used for the first period. However, this might vary with the objective of the program.

## Conclusion

Time integration of environmental data over a year from a set of sampling stations representing a given area can be used in environmental monitoring surveys to compare inter-annual differences and detect long-term trends with high precision. Such a data treatment will eliminate the problem of non-normal distribution and autocorrelation between samples in time series. The use of a simple allocation model helps the user to optimize the precision-cost relationship in such a survey.
